# Teachers' perspectives on the application of technology in mathematics education in primary schools: A dataset from Vietnam

**DOI:** 10.1016/j.dib.2025.111473

**Published:** 2025-03-15

**Authors:** Quoc Anh Vuong, Dien Thi Bui, Hue Thi Thu Dang, Anh Vinh Le, Duc Lan Do

**Affiliations:** The Vietnam National Institute of Educational Sciences, 101 Tran Hung Dao Street, Hoan Kiem District, Hanoi 100000, Vietnam

**Keywords:** Educational technology, Teaching mathematics, School mathematics, Primary education

## Abstract

This dataset offers insights into the integration of technology in primary school mathematics education, as perceived and evaluated by primary school teachers in Vietnam. Based primarily on the Technology Acceptance Model (TAM), it covers five key aspects from teachers’ perspectives: (a) Teacher background information, including gender, teaching experience, qualifications, and location; (b) Teachers' views on the role of technology, encompassing AR technology and its application in mathematics education; (c) Practical implementation of technology in primary school mathematics classrooms, detailing tools used, instructional methods, assessment practices, frequency of implementation, and evaluation; (d) Teachers' self-assessment of technology integration effectiveness, including high-tech applications like AR technology in primary school mathematics education; and (e) Teaching conditions related to technology integration and teacher readiness to prepare and utilize technology in mathematics teaching. The survey, conducted online via Google Forms from October to December 2023, involved 11,811 primary school teachers from ten provinces in Vietnam. This dataset aims to provide valuable insights for policymakers and educational administrators to understand the current landscape of technology use in mathematics education. It seeks to inform policy decisions and initiatives related to technology integration, including AR technology in mathematics teaching, and supports efforts to enhance the quality of mathematics instruction. Furthermore, the dataset contributes to evidence-based interventions aimed at supporting teachers' professional development in applying technology in mathematics education. Educational managers and researchers can leverage this dataset to gain insights into pedagogical improvements such as teacher training and policy formulation related to technology integration in mathematics education. Additionally, this dataset can assist educational technology developers in understanding teachers’ needs, readiness, actual use, and practices to develop technology applications for mathematics teaching. Overall, this dataset is valuable in providing an overview of technology applications in mathematics education, supporting educators and policymakers with evidence-based strategies to enhance technology integration, and guiding educational technology developers in aligning future developments with teachers' practical needs and capabilities.

Specifications Table

**Table:** 

Subject	Social sciences
Specific subject area	*Education* *Pedagogy* *Educational technology* *Teaching Mathematics*
Type of data	Table, Figure, Raw, Analysed
Data collection	The data collection process involved administering a questionnaire developed based on the TAM model [3,6]. An online survey was created using Google Forms, and the survey link was distributed to primary school teachers through the educational management systems of the ten Departments of Education and Training. Responses from teachers were then imported into an Excel spreadsheet for initial data organization and cleaning. Subsequently, statistical analysis was conducted using SPSS Version 26 to identify trends and patterns within the dataset.
Data source location	Institution: The Vietnam National Institute of Educational Sciences City/Town/Region: Tuyen Quang, Hanoi, Thai Binh, Can Tho, Thua Thien Hue, Ninh Thuan, Dak Lak, Kon Tum, Dong Thap, and Ca Mau Country: Vietnam Latitude and longitude (and GPS coordinates, if possible) for collected samples/data: Tuyen Quang: 21.7830693° N, 105.1514514 ° E Hanoi: 21.0227384 ° N, 105.8163641° E Thai Binh: 20.4529365° N, 106.3034525° E Thua Thien Hue: 16.368564° N, 107.274251° E Ninh Thuan: 11.7378731° N, 108.5624599° E Dak Lak: 12.7761524° N, 105.7383303° E Kon Tum: 14.3428533° N, 107.8870684° E Can Tho: 10.0341844° N, 105.7163706° E Dong Thap: 10.5543054° N, 105.234831° E Ca Mau: 9.175231° N, 104.875322° E
Data accessibility	Repository name: Mendeley Data Data identification number: 10.17632/khsnjy2hn2.1 Direct URL to data: https://data.mendeley.com/datasets/khsnjy2hn2/1
Related research article	

## 1 Value of the Data

• The dataset offers a comprehensive database of primary school teachers' assessments regarding the use of technology in teaching Mathematics in Vietnamese primary schools.
• The dataset provides a comprehensive repository of primary school teachers' evaluations regarding the integration of technology in Mathematics teaching across Vietnamese primary schools.
• Insights from the dataset can inform evidence-based policies and interventions aimed at supporting educational managers and teachers in enhancing the quality of mathematics education and other subjects through effective technology integration.
• The dataset contributes empirical evidence to the field of technology implementation, benefiting future research endeavours. Vietnamese and international researchers and educators can utilize this data to explore appropriate technology applications, develop tailored tools and programs based on teachers' needs, and propose strategies to address challenges and optimize the effectiveness of technology applications.
• The dataset can serve as a reference for educational technology developers seeking to understand the practical needs and usage realities of teachers, particularly in developing countries, to create educational technology platforms that support teachers in teaching Mathematics.
• Additionally, the dataset can inform policy makers on strategies to promote STEM programs in education, enhancing the quality of STEM subjects through technology use.

## 2 Background

The integration of technology in education, particularly in mathematics instruction, has garnered global attention due to its potential to enhance learning outcomes in what is often perceived as a challenging subject [1,2,7]. Mathematics holds a pivotal role in STEM (Science, Technology, Engineering, and Mathematics) education, underscoring the importance of effective instructional strategies, especially in early education stages [5].

Given the dynamic landscape of educational technology and the necessity for evidence-based practices [7,8], this research aims to capture primary school teachers' perspectives on technology integration in mathematics education. The theoretical framework for this dataset is grounded in the Technology Acceptance Model (TAM) [3,6], which elucidates user acceptance and use of technology. Specifically, it explores perceived usefulness (PU), perceived ease of use (PEU), attitude toward use (AT), behavioral intention to use (BI), and actual usage behavior. The dataset encompasses insights from teachers across various domains: (A) Background Information, (B) Teachers' Perspectives on Educational Technology, (C) Practical Application of Technology in Teaching Mathematics, (D) Evaluation of Technology Effectiveness in Teaching Mathematics, and (E) Infrastructure Conditions and Teachers' Readiness to Apply Technology. Additionally, it addresses modern issues like Augmented Reality (AR) in mathematics education, examining teacher readiness and attitudes toward technological advancements [4].

This dataset serves as a valuable resource for understanding teachers' viewpoints, experiences, challenges, and opportunities related to technology integration in primary mathematics classrooms. It contributes empirical evidence to ongoing research efforts aimed at improving mathematics instruction through effective technology integration. Furthermore, it informs policy decisions and educational interventions geared toward enhancing the quality and efficacy of mathematics education in Vietnamese primary schools. This dataset provides substantial contributions to the broader discourse on technology integration in mathematics education, facilitating further investigations and initiatives in this field.

## 3 Data Description

The dataset, available on Mendeley Data [9], provides valuable insights into the utilization of technology in teaching Mathematics at the primary school level. It includes a comprehensive questionnaire comprising 46 items, with raw data consisting of 11,811 responses from 10 provinces in Vietnam. The raw data is structured into two sheets: Sheet 1 serves as the codebook, detailing each question's content and corresponding coded responses, while Sheet 2 contains the raw data with teachers' answers encoded according to the codebook. The questionnaire is organized into five main sections:

• Demographic Information: This section, detailed in Table 1, includes school location, age, gender, teaching experience, and teacher qualifications. These demographic details can be analyzed for correlations with other questionnaire items. It consists of 6 items, ranging from Question 1 to Question 6 in the codebook.

**Table 1:** General information of participating teachers by location, gender, and teaching experience.

Total 11811	School located		Gender		Teaching experience				
	Urban area	Rural area	Male	Female	Less than 5 years	From 5 to under 10 years	From 10 to under 15 years	From 15 to under 20 years	20 years or more
	5173 (43.8 %)	6638 (56.2 %)	3102 (26.3 %)	8709 (73.7 %)	1499 (12.7 %)	1303 (11 %)	1522 (12.9 %)	1229 (10.4 %)	6258 (53 %)
• Teachers' Perspectives on Educational Technology: This section, comprising 3 items in Section B of the codebook, explores teachers' evaluations of the role of educational technology in primary school Mathematics education, including their views on the necessity and familiarity with advanced technologies like augmented reality (AR) applications/software.
• Practical Application of Technology in Teaching Mathematics: This section, with 10 items, delves into teachers' actual use of educational technology in teaching Mathematics. It covers frequency of technology application (Question 4), usage of specific technology applications (Questions 5.1 to 5.6), and the use of technology for teaching and assessing students' learning outcomes in Mathematics (Questions 6.1 to 6.3).
• Evaluation of Technology Effectiveness in Teaching Mathematics: This section contains 11 items and assesses teachers' opinions on the benefits of educational technology for primary school Mathematics education. It includes their views on its impact on students' interest in math, performance, communication skills, cooperation skills, autonomy and self-regulation skills, and problem-solving and creativity skills (Questions 7.1 to 7.5). Additionally, it covers the perceived benefits for teachers in terms of class organization, active teaching methods, and effective student assessment (Questions 8.1 to 8.3). Teachers familiar with AR applications/software also answer Questions 9 to 11, evaluating the effectiveness and desire for AR technology in teaching primary school Mathematics.
• Infrastructure Conditions and Teachers' Readiness: This final section includes 16 items in Section E, focusing on the availability of digital equipment and infrastructure supporting technology application both at home (Questions 12.1 to 12.4) and in schools (Questions 13.1 to 13.5). It also covers teachers' IT skills (Question 14), their self-assessed confidence in applying technology in Math lessons (Question 15), and perceived difficulties in using educational technology in teaching Mathematics at the primary school level (Questions 16.1 to 16.5).

Table 1, Table 2, Table 3, Table 4 in the dataset illustrate various findings, including differences in urban and rural teachers' perceptions of educational technology's role, frequency of technology use in teaching mathematics, regional variations in desire for AR technology application, and self-assessment of technology application confidence among teachers Table 5.

**Table 2:** Differences between urban and rural teachers' evaluation of the importance of educational technology in primary school teaching.

Independent Samples Test										
		Levene's Test for Equality of Variances		t-test for Equality of Means						
		F	Sig.	t	df	Sig. (2-tailed)	Mean Difference	Std. Error Difference	95 % Confidence Interval of the Difference	
									Lower	Upper
How do you evaluate the role of educational technology in teaching and learning at the primary level?	Equal variances assumed	114.763	.000	12.585	11809	.000	.160	.013	.135	.185
	Equal variances not assumed			12.552	11001.531	.000	.160	.013	.135	.185

**Table 3:** Frequency of technology application in primary school mathematics teaching.

	Mean	SD	Never	Rarely	Occasionally	Frequently	Always
Specialized software on computers	3.48	.928	415 (3.5 %)	982 (8.3 %)	4299 (36.4 %)	4727 (40 %)	1388 (11.8 %)
Online teaching support applications	2.73	1.041	1821 (15.4 %)	2508 (21.2 %)	4870 (41.2 %)	2206 (18.7 %)	406 (3.4 %)
Specialized apps on smartphones/tablets	2.52	1.144	3002 (25.4 %)	2541 (21.5 %)	3778 (32 %)	2108 (17.8 %)	382 (3.2 %)
Learning management systems	2.91	1.153	1833 (15.5 %)	2098 (17.8 %)	3935 (33.3 %)	3145 (26.6 %)	800 (6.8 %)
General software/applications/games	2.65	1.096	2284 (19.3 %)	2625 (22.2 %)	4318 (36.6 %)	2145 (18.2 %)	439 (3.7 %)
Hightech applications like AR in math lessons	1.54	1.078	9125 (77.3 %)	454 (3.8 %)	1111 (9.4 %)	814 (6.9 %)	307 (2.6 %)

**Table 4:** Regional differences in teachers' desire for ar technology application in primary school mathematics teaching.

**Group Statistics**										
	School located		N		Mean		Std. Deviation		Std. Error Mean	
What is your level of desire for the application of AR technology in teaching primary school mathematics	City		1713		3.95		.858		.021	
	Countryside		973		3.68		.908		.029	
**Independent Samples Test**										
		Levene's Test for Equality of Variances		t-test for Equality of Means						
		F	Sig.	t	df	Sig. (2-tailed)	Mean Difference	Std. Error Difference	95 % Confidence Interval of the Difference	
									Lower	Upper

What is your level of desire for the application of AR technology in teaching primary school mathematics	Equal variances assumed	40.335	.000	7.860	2684	.000	.276	.035	.207	.345
	Equal variances not assumed			7.738	1926.236	.000	.276	.036	.206	.347

**Table 5:** Regional variations in teachers' self-assessed confidence in applying technology in math lessons.

	**Group Statistics**				
	School located	N	Mean	Std. Deviation	Std. Error Mean
How do you evaluate your confidence in applying technology in math lessons	City	5173	3.99	.700	.010
	Countryside	6638	3.70	.689	.008


**Independent Samples Test**										
		Levene's Test for Equality of Variances		t-test for Equality of Means						
		F	Sig.	t	df	Sig. (2-tailed)	Mean Difference	Std. Error Difference	95 % Confidence Interval of the Difference	
									Lower	Upper
How do you evaluate your confidence in applying technology in math lessons	Equal variances assumed	200.572	.000	22.588	11809	.000	.291	.013	.265	.316
	Equal variances not assumed			22.541	11025.268	.000	.291	.013	.265	.316

This dataset serves as a crucial resource for understanding teachers' perspectives and practices regarding technology in primary school mathematics education, contributing to ongoing efforts to enhance educational quality through evidence-based strategies and interventions.

## 4 Experimental Design, Materials and Methods

### 4.1 Data tools

The tool is grounded in the Technology Acceptance Model (TAM) and covers five main aspects from teachers’ perspectives:

• Teacher Background Information: Includes demographic details such as gender, teaching experience, qualifications, and geographical location.
• Teachers' Views on the Role of Technology: Explores perceptions of educational technology, including advanced applications like Augmented Reality (AR) in mathematics education.
• Practical Implementation of Technology: Details the tools used, instructional methods, assessment practices, frequency of technology implementation, and evaluation metrics in primary school mathematics classrooms.
• Teachers' Self-assessment of Technology Integration Effectiveness: Evaluates the perceived effectiveness of technology integration, particularly high-tech applications such as AR technology, in primary school mathematics education.
• Teaching Conditions Related to Technology Integration: Assesses teachers' readiness and the educational environment's readiness for technology integration in mathematics teaching.

The survey was administered online using Google Forms from October to December 2023, involving 11,811 primary school teachers across ten provinces in Vietnam.

### 4.2 Experimental Design

#### 4.2.1 Survey Design and Administration

The survey instrument was designed to collect quantitative data aligned with the research objectives. It comprised structured questions distributed across the five thematic areas mentioned above. The survey items were crafted to capture detailed insights into teachers’ perspectives on technology use in mathematics education, focusing on perceptions, practices, and challenges.

### 4.3 Sampling Strategy

The study targeted teachers in grades 1 through 5 at the primary school level during the 2023-2024 academic year. Sampling aimed to ensure representation from diverse geographic regions and locality types in Vietnam, including urban, rural, and mountainous areas. Participants were recruited through educational networks and professional associations. The online survey link was distributed widely among primary school teachers in Vietnam. Respondents accessed the survey via Google Forms, which allowed for efficient data collection and management.

### 4.4 Pilot Testing

Before widespread distribution, the questionnaire underwent pilot testing with 86 teachers. Feedback from the pilot phase was used to revise the questionnaire for clarity and to optimize the number of items. The final questionnaire demonstrated high internal consistency, with a Cronbach's alpha value of 0.973.

### 4.5 Data Collection

#### 4.5.1 Geographical Representation

Data collection occurred in 10 provinces selected for their diverse geographic representation across Northern, Central, and Southern Vietnam. These provinces included Hanoi, Can Tho, Ca Mau, Dak Lak, Dong Thap, Kon Tum, Ninh Thuan, Thai Binh, Thua Thien Hue, and Tuyen Quang.

#### 4.5.2 Participant Demographics

Teachers from these provinces, encompassing varied locality types, ages, and work experiences, participated in the survey.

#### 4.5.3 Data Analysis

The study received a total of 12,232 responses. After excluding 421 responses due to missing data, a final dataset of 11,811 responses was analyzed using IBM SPSS Version 26.

## Limitations

This study utilized an online questionnaire. While online questionnaires with Google Forms have several strengths, such as allowing respondents to complete surveys at their own convenience and reaching a broad audience regardless of geographical location, they also have some limitations. These forms are accessible on multiple devices and can offer anonymity to respondents, encouraging more honest and candid responses. However, self-selection bias can occur, where only those interested in the topic or with strong opinions are likely to respond. However, these limitations do not substantially undermine the accuracy and credibility of the data, given the survey's large sample size. There is also a higher risk of incomplete responses or rapid, non-thoughtful answers due to the ease of clicking through the form. This limitation can be minimized through data cleaning.

## Ethics Statement

The study adhered to ethical guidelines for research involving human subjects, ensuring informed consent, confidentiality, and voluntary participation. Institutional review board approval was obtained prior to data collection. In details, the Ethics Committee of the Vietnam National Institute of Educational Sciences granted approval and oversaw the procedures for conducting this research (ethics approval number: B2023.VKG.25.GRANTED). The data collection process strictly adhered to the ethical guidelines and regulations set forth by the committee. Teachers were fully informed about the research objectives and provided their consent before completing the questionnaire. Measures were taken to ensure the anonymity and confidentiality of participants throughout the research process.

## Declaration of generative AI and AI-assisted technologies in the writing process

During the preparation of this work, the authors used ChatGPT 3.5 for grammar and language editing of our writing. After utilizing this tool/service, the authors reviewed and edited the content as necessary, taking full responsibility for the publication's content.

## CRediT authorship contribution statement

**Quoc Anh Vuong:** Conceptualization, Methodology, Software, Supervision, Data curation, Validation. **Dien Thi Bui:** Investigation, Methodology, Writing – original draft, Writing – review & editing. **Hue Thi Thu Dang:** Formal analysis, Writing – review & editing. **Anh Vinh Le:** Resources, Supervision, Project administration. **Duc Lan Do:** Visualization, Writing – review & editing.

## Data Availability

Mendeley DataDataset on Teachers' Perspectives on the Application of Technology in Teaching Mathematics in Primary Schools (Original data).

## Appendix Supplementary materials

Image, application 1
