# Supplementary material for: Teachers' perspectives on the application of technology in mathematics education in primary schools: A dataset from Vietnam
Source: Data Brief. 2025 Mar 15;60:111473. doi: 10.1016/j.dib.2025.111473 (PMC11985059; doi:10.1016/j.dib.2025.111473)
Supplement: Supplementary file 1 [file mmc1.docx]

**Online Consent Form for Teachers**

**Title of the Research Project:** *Assessing the Effectiveness of Augmented Reality (AR) Application in Primary School Mathematics Education*
**Project Code:** B2023-VKG-25
**Ethics Approval Number:** B2023.VKG.25.GRANTED
**Conducted by:** Vietnam National Institute of Educational Sciences
**Funding Authority:** Vietnam Ministry of Education and Training (Decision 2036/QĐ-BGDĐT)

**Dear Teacher,**

We warmly invite you to participate in our research project exploring the integration of Augmented Reality (AR) technology in primary school mathematics education. This research aims to evaluate the effectiveness of AR applications in teaching mathematics and identify strategies to support teachers in utilizing technology in the classroom.

Your participation involves completing a questionnaire about your experiences, perspectives, and practices related to technology integration in mathematics teaching. The survey is voluntary, and you may withdraw at any time without consequences.

All responses will be kept strictly confidential and anonymous. The data collected will be used solely for research purposes to inform educational policies and improve teacher support systems.

**Participation Agreement**

**Do you agree to participate in this research?**

🔘 **Yes, I agree to participate in this research and allow my responses to be used anonymously for academic and policy-related purposes.** *(Leads to the survey link.)*

🔘 **No, I do not agree to participate.** *(Ends the form.)*

We sincerely appreciate your time and valuable contribution to this research. Thank you for helping us improve technology integration in primary education.

**Best regards,**
**The Research Team**
Vietnam National Institute of Educational Sciences
